# Supplementary material for: Novel Treatment Strategies for Low-Risk Metastatic Castration-Sensitive Prostate Cancer
Source: Cancers (Basel). 2024 Sep 19;16(18):3198. doi: 10.3390/cancers16183198 (PMC11430633; doi:10.3390/cancers16183198)
Supplement: Supplementary file 1 [file cancers-16-03198-s001.zip › cancers-3118463-supplementary.pdf]

Supplementary Table S1. Univariate and multivariate analyses of TTCR in LATITUDE low risk mCSPC (serum markers at 4W after ADT).

			Univariate		Multivariate	
		n	HR (95% CI)	*P*	HR (95% CI)	*P*
PSA reduction rate, ng/mL	≥ 95%	13	1.37 (0.57-3.28)	0.48	1.12 (0.32-3.83)	0.86
	< 95%	24				
LDH at 12W after ADT, IU/L	<250 or reduction rate ≥ 30%	25	0.44 (0.057-3.43)	0.44	0.35 (0.43-2.90)	0.33
	≥250 and reduction rate < 30%	2				
ALP at 12W after ADT, IU/L	<350 or reduction rate ≥ 30%	15	1.12 (0.43-2.93)	0.82	0.94 (0.32-2.77)	0.91
	≥350 and reduction rate < 30%	12				

TTCR: time to castration resistance; mCSPC: metastatic castration-sensitive prostate cancer; ADT: androgen deprivation therapy; PSA: prostate-specific antigen; LDH: lactate dehydrogenase; ALP: alkaline phosphatase; HR: hazard ratio; CI: confidence interval.

Supplementary Table S2. Univariate and multivariate analyses of TTCR in CHAARTED low volume mCSPC (serum markers at 4W after ADT).

			Univariate		Multivariate	
		n	HR (95% CI)	*P*	HR (95% CI)	*P*
PSA reduction rate, ng/mL	≥ 95%	16	1.37 (0.61-3.08)	0.45	1.01 (0.33-3.03)	0.99
	< 95%	23				
LDH at 12W after ADT, IU/L	<250 or reduction rate ≥ 30%	29	0.37 (0.048-2.83)	0.34	0.37 (0.045-3.03)	0.35
	≥250 and reduction rate < 30%	2				
ALP at 12W after ADT, IU/L	<350 or reduction rate ≥ 30%	13	1.28 (0.52-3.19)	0.59	1.24 (0.44-3.54)	0.68
	≥350 and reduction rate < 30%	15				

TTCR: time to castration resistance; mCSPC: metastatic castration-sensitive prostate cancer; ADT: androgen deprivation therapy; PSA: prostate-specific antigen; LDH: lactate dehydrogenase; ALP: alkaline phosphatase; HR: hazard ratio; CI: confidence interval.

Supplementary Table S3. Univariate and multivariate analyses of TTCR in Canazawa low risk mCSPC (serum markers at 4W after ADT).

			Univariate		Multivariate	
		n	HR (95% CI)	*P*	HR (95% CI)	*P*
PSA reduction rate, ng/mL	≥ 95%	27	0.78 (0.43-1.40)	0.40	0.68 (0.36-1.29)	0.24
	< 95%	40				
LDH at 12W after ADT, IU/L	<250 or reduction rate ≥ 30%	60	0.44 (0.11-1.84)	0.26	0.45 (0.10-1.95)	0.29
	≥250 and reduction rate < 30%	4				
ALP at 12W after ADT, IU/L	<350 or reduction rate ≥ 30%	36	1.58 (0.87-2.86)	0.13	1.26 (0.66-2.40)	0.48
	≥350 and reduction rate < 30%	27				

TTCR: time to castration resistance; mCSPC: metastatic castration-sensitive prostate cancer; ADT: androgen deprivation therapy; PSA: prostate-specific antigen; LDH: lactate dehydrogenase; ALP: alkaline phosphatase; HR: hazard ratio; CI: confidence interval.
